# Body mass index and the risk of abdominal aortic aneurysm presence and postoperative mortality: a systematic review and dose-response meta-analysis

**DOI:** 10.1097/JS9.0000000000001125

**Published:** 2024-02-05

**Authors:** Yihao Wu, Hao Zhang, Deying Jiang, Fanxing Yin, Panpan Guo, Xiaoxu Zhang, Jian Zhang, Yanshuo Han

**Affiliations:** a School of Life and Pharmaceutical Sciences, Dalian University of Technology, Panjin; b Department of Vascular Surgery, Central Hospital of Dalian University of Technology Dalian; c Department of Vascular Surgery, The First Hospital of China Medical University, Shenyang, People’s Republic of China

**Keywords:** abdominal aortic aneurysm (AAA), body mass index (BMI), dose-response meta-analysis, obesity paradox, postoperative mortality

## Abstract

**Background:**

The clinical data regarding the relationships between BMI and abdominal aortic aneurysm (AAA) are inconsistent, especially for the obese and overweight patients. The aims of this study were to determine whether obesity is associated with the presence of AAA and to investigate the quantitative relationship between BMI and the risk of AAA presence and postoperative mortality.

**Materials and methods:**

PubMed, Web of Science, and Embase databases were used to search for pertinent studies updated to December 2023. The pooled relative risk (RR) with 95% CI was estimated by conventional meta-analysis based on random effects model. Dose-response meta-analyses using robust-error meta-regression (REMR) model were conducted to quantify the associations between BMI and AAA outcome variables. Subgroup analysis, sensitivity analysis, and publication bias analysis were performed according to the characteristics of participants.

**Results:**

Eighteen studies were included in our study. The meta-analysis showed a higher prevalence of AAA with a RR of 1.07 in patients with obesity. The dose-response meta-analysis revealed a nonlinear relationship between BMI and the risk of AAA presence. A ‘U’ shape curve reflecting the correlation between BMI and the risk of postoperative mortality in AAA patients was also uncovered, suggesting the ‘safest’ BMI interval (28.55, 31.05) with the minimal RR.

**Conclusions:**

Obesity is positively but nonlinearly correlated with the increased risk of AAA presence. BMI is related to AAA postoperative mortality in a ‘U’ shaped curve, with the lowest RR observed among patients suffering from overweight and obesity. These findings offer a preventive strategy for AAA morbidity and provide guidance for improving the prognosis in patients undergone AAA surgical repair.

## Graphical abstract

**Figure:**
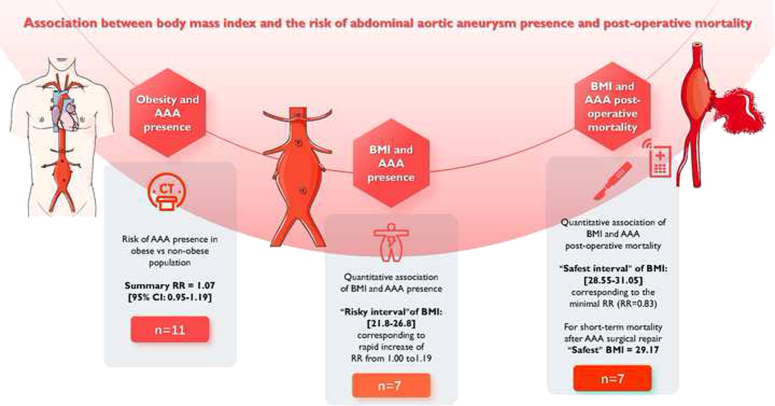

The graphic abstract that provides a visual summary of the aims, methods, and major conclusions of the paper.

## Introduction

Highlights

- The first meta-analysis revealing the quantitative relationship between BMI and the risk of abdominal aortic aneurysm (AAA) presence and postoperative mortality.
- The nonlinear relationship between BMI and the risk of AAA presence.
- A ‘U’ shape curve reflecting the correlation between BMI and the risk of postoperative mortality in AAA patients.
- Offer novel insights into the prevention and prognosis of AAA among specific populations.

Abdominal aortic aneurysm (AAA) is an age-related vascular disease featured with the pathological dilatation and the consistent weakening of the aortic wall^1^. AAA occurs in 9% of adults aged of ≥65 years and is responsible for a significant number of deaths in Western countries^2^. AAA is also an important cause of sudden death in older adults’ population due to aortic rupture and other cardiovascular complications. Although the etiology of AAA is partly unraveled, AAA is considered to be resulted from several risk factors including older age, smoking, hypertension, hyperlipidemia, atherosclerosis, and family history^3,4^. However, the role of obesity as a risk factor for AAA was previously discussed but still remains understudied and poorly understood for a long period of time. Golledge *et al*.^5^ identified that measures of obesity (i.e. waist circumference, waist-to-hip ratio, and serum resistin concentration) were independently associated with increased risk of AAA in their cohort study. The review article by Cronin *et al*.^6^ suggested that BMI was positively associated with AAA presence. However, Takagi and Umemoto^7^ stated that obesity, in terms of BMI, appears to be unassociated with AAA presence in their meta-analysis. Therefore, in 2020, based on this controversy, Eckstein and Maegdefessel^8^ appealed to researchers and surgeons to pay more attention to this issue and try to reasonably link obesity with AAA development.

Obesity, defined as an increase in fat at a sufficient level to cause adverse health consequences, is usually diagnosed by anthropometric measurements of BMI, which is calculated as weight in kilograms divided by the square of height in meters (kg/m^2^)^9^. Some unexpected results have been implied in recent studies about the relationship between obesity/BMI and cardiovascular diseases, indicating a phenomenon called ‘obesity paradox’. Typically, the obesity paradox refers to the finding of a lower mortality rate and survival benefit for overweight or obese people in some stages of cardiovascular and peripheral vascular diseases^10^. For example, in the analysis of long-term survival among patients with peripheral artery disease, the lowest mortality rates were actually observed in patients with obesity^11^. Another cohort study completed by Davenport *et al*.^12^ reported that mildly obese patients have reduced comorbid illness and mortality for 30-day outcomes after vascular surgery compared to normal-class patients. Currently, different mechanisms and biological hypotheses have been postulated to support and explain the existence of obesity paradox. For instance, the obesity paradox may be related to the lipid metabolism and cytokines production by adipose tissue^13^. Several adipokines produced by adipose tissue have shown to be cardioprotective and to exert a variety of beneficial effects on cardiovascular function^14^.

Currently, the most efficient way to treat AAA is surgical repair, which contains two major types: endovascular aneurysm repair (EVAR) and open surgical repair (OSR). Notably, the ‘obesity paradox’ was also found in the outcomes of AAA surgical repair in patients with or without obesity. Naiem *et al*.^15^ demonstrated that patients with obesity have lower 30-day mortality after EVAR compared with patients unaffected by obesity. Besides, Zonneveld *et al*.^16^ also performed a relevant meta-analysis, suggesting that obesity is not a risk factor for short-term mortality after AAA repair compared to nonobesity and actually patients with obesity suffer less from cardiac complications. However, these previous meta-analyses only provided general conclusions about the relationship between obesity/BMI and the outcomes of AAA surgical repair but failed to dig out any quantitative associations between them. Hence, we believe that conducting a new meta-analysis is necessary to clarify the relationship between obesity/BMI and AAA.

Nowadays, the methodological advances in the area of dose-response meta-analysis provide powerful analytical tools to achieve our goals. To date, there has not been any study that examines the quantitative relationship between BMI and the risk of AAA presence and postoperative mortality based on cohort studies. Therefore, aiming at further exploring the ‘obesity paradox’ in AAA, we conducted this updated overall and dose-response meta-analysis. The main purposes of this study were as follows: (1) to determine whether there is an association between obesity and the risk of AAA presence; (2) to clarify the quantitative relationship between BMI and the risk of AAA presence by dose-response meta-analysis; and (3) to explore the quantitative association between BMI and the risk of mortality after AAA surgical repair by dose-response meta-analysis.

## Methods

The study protocol was registered in the international prospective register of systematic reviews. Our systematic review/meta-analysis has been reported in line with the Preferred Reporting Items for Systematic reviews and Meta-Analyses (PRISMA, Supplemental Digital Content 1, http://links.lww.com/JS9/B787, Supplemental Digital Content 2, http://links.lww.com/JS9/B788) and Assessing the methodological quality of systematic reviews (AMSTAR, Supplemental Digital Content 3, http://links.lww.com/JS9/B789) guidelines^17,18^.

### Data sources and search strategy

The two investigators independently searched the PubMed, Web of Science, and Embase databases for relevant studies examining the associations between obesity or BMI and the risk of AAA presence or postoperative mortality on 11 December 2023, without the restriction of language and publication date for eligible studies. In addition, reference lists of the retrieved articles and reviews on the subject were also manually evaluated to identify any other relevant published articles. We combined keywords and MeSH terms in our search and the detailed search strategy for each database is provided in SDC Table 1 (Supplemental Digital Content 4, http://links.lww.com/JS9/B790).

### Eligibility criteria and study selection

The inclusion criteria were in accordance with the Population, Intervention/Exposure, Control, Outcomes, and Study design (PICOS) framework. Taking the dose-response meta-analysis of BMI and its association with AAA presence as an example, PICOS framework was applied based on the following selection criteria:

Population: Patients suffered from AAA;

Intervention/Exposure: the BMI outcomes had to be reported in both exposure and reference groups (means or medians) or clearly defined according to WHO classification criteria^19^;

Control: healthy people without AAA;

Outcomes: AAA presence;

Study design: cohort study, case–control study, and cross-sectional study.

For the dose-response meta-analysis of BMI and the risk of AAA postoperative mortality, the population was defined as patients underwent AAA surgical repair and the outcomes were fatality followed by AAA surgical repair.

In the process of title and abstract screening, we eliminated studies which failed to investigate the association between cardiovascular risk factors and AAA. Review articles, conference, and meeting abstracts were also excluded.

In full-text review, the eligible studies were included as the following general criteria: (1) the study design must be an epidemiological study design (i.e. cohort study, case–control study, or cross-sectional study); (2) clearly defining the intervention or exposure (BMI/obesity) and the outcomes of interest (AAA presence or postoperative mortality); (3) reporting the multivariate-adjusted relative risk (RR) or odds ratio (OR) or hazard ratio (HR) and the corresponding 95% CI for ≥2 categories of BMI; (4) if study populations overlapped, we selected the one with the most updated, largest sample size; (5) control group had to be from the same geographic region, and the same exposure measurement methods had to exist between cases and controls. Overall, study selection was performed by two independent reviewers, and any discrepancies were resolved through discussion with a third reviewer.

### Data extraction and quality assessment

Data extraction was conducted by one author and double-checked by a second author. For the meta-analysis of BMI and the risk of AAA presence, the following characteristics were extracted from each study: first author; year of publication; the country where the study was conducted; the cohort’s name (if applicable); study design; number of controls and AAA cases; gender of controls and AAAs; age of controls and AAAs; exposure level (BMI groups with mean); multivariable adjusted OR/RR/HR with 95% CI; adjustment confounders. For the meta-analysis of BMI and AAA postoperative mortality, similar information was extracted from each study in addition to type of surgical repair; length of follow-up and outcomes. Among our included studies, postoperative mortality was yielded by follow-up for 7 years at maximum. Short-term mortality was defined as all-cause mortality on perioperative 30-day outcomes and long-term mortality was defined as all-cause mortality on postoperative follow-up (>30 days but <7 years) outcomes. The extracted data characteristics were summarized in Tables 1–4.

**Table 1:** Data of subjects enrolled in studies evaluating BMI and its association with AAA presence.

									Age				
					Number of participants		Men *n* (%)		AAA		Controls		
No.	Author	Year	Study type	Country	AAA	Controls	AAA	Controls	Mean	SD	Mean	SD	Multivariable adjusted OR/RR/HR
1	O. Stackelberg *et al*.^20^	2012	Cohort study	Sweden	492	NA	492 (100)	NA	74.0	7.0	NA	NA	✓
2	O. Stackelberg *et al*.^20^	2012	Cohort study	Sweden	105	NA	0 (0)	0 (0)	76.1	6.9	NA	NA	✓
3	Lu Wang *et al.* ^21^	2017	Cohort study	USA	471	25083	471 (100)	25083 (100)	70.8	7.4	65.4	9.0	✓
4	K. Craig Kent *et al.* ^22^	2010	Cohort study	USA	23446	3033009	79.34	34.93	62.98	NA	71.10	NA	✓
5	Kevin C. Chun *et al.* ^23^	2014	Cohort study	USA	469	5673	465 (99.1)	5653 (99.6)	NA	NA	NA	NA	✓
6	Daniel R. Wong *et al.* ^24^	2007	Cohort study	USA	376	38976	376 (100)	38976 (100)	NA	NA	NA	NA	✓
7	Kelli L. Summers *et al.* ^25^	2020	Cohort study	USA	267	9190	208 (77.9)	4272 (46.5)	NA	NA	NA	NA	✓
8	Eiman Jahangir *et al.* ^26^	2015	Cohort study	USA	158	6528	158 (100)	6528 (100)	67.5	5.3	64.4	5.6	✓
9	Eiman Jahangir *et al.* ^26^	2015	Cohort study	USA	123	11692	0 (0)	0 (0)					✓
10	Linn Nyrønning *et al.* ^27^	2019	Cohort study	Norway	622	58514	461 (74.1)	27693 (47.3)	NA	NA	NA	NA	✓
11	Toril Rabben *et al.* ^28^	2021	Cohort study	Norway	330	12480	330 (100)	12480 (100)	NA	NA	NA	NA	✓

**Table 2:** Data of subjects enrolled in studies evaluating BMI and its correlation with the postoperative mortality.

							Age			
No.	Author	Year	Study type	Country	Number of patients	Men (%)	Mean (SD)	Surgical repair type	Length of follow-up	Multivariable adjusted OR/RR/HR
1	Michael S. Miller *et al.* ^29^	2019	Cohort study	USA	492	411 (95.8)	NA (NA)	EVAR	30 days/9 years	✓
2	Kristina A Giles *et al.* ^30^	2010	Cohort study	USA	2097	73.8	71.5 (8.5)	OSR	30 days	✓
3	Kristina A Giles *et al.* ^30^	2010	Cohort study	USA	3358	82.8	74.1 (8.4)	EVAR	30 days	✓
4	William P. Shutze Sr *et al.* ^31^	2018	Cohort study	USA	336	278 (82.7)	73.7 (8.7)	EVAR	5 years	✓
5	Joshua K. Kays *et al.* ^32^	2018	Cohort study	USA	505	467 (92.5)	69.7 (8.6)	OSR and EVAR	30 days	✓
6	Athanasios Saratzis *et al.* ^33^	2014	Cohort study	UK	159	144 (90.6)	69.0 (9.0)	EVAR	34±13 months	✓
7	Timothy C. Huber *et al.* ^34^	2019	Cohort study	USA	407	343 (84.3)	72.2 (9.0)	EVAR	39±33.5 months	✓

**Table 3:** Characteristics of individual studies on BMI and the risk of AAA presence.

					Number of participants				
No.	Author	Year	Study type	Country	AAA	Controls	BMI groups (mean)	Relative risk (95% CI) in multivariate regression model	Adjustment for confounders
1	O. Stackelberg *et al*.^20^	2012	Cohort study	Sweden	492 (Male)	NA	<25 (23.0) 25–29.9 (26.8) ≥30 (31.6)	1.00 (1.00–1.00) 0.86 (0.69–1.07) 0.88 (0.60–1.28)	Age, educational level, smoking, alcohol consumption, and dichotomous for diabetes, hypertension, hypercholesterolemia and cardiovascular disease
2	O. Stackelberg *et al*.^20^	2012	Cohort study	Sweden	105 (Female)	NA	<25 (23.0) 25–29.9 (26.8) ≥30 (31.6)	1.00 (1.00–1.00) 0.81 (0.50–1.33) 0.71 (0.33–1.55)	Age, educational level, smoking, alcohol consumption, and dichotomous for diabetes, hypertension, hypercholesterolemia and cardiovascular disease
3	Lu Wang *et al.* ^21^	2017	Cohort study	USA	471	25 083	<25 (22.5) 25–29.9 (27.5) ≥30 (32.5)	1.00 (1.00–1.00) 1.30 (1.06–1.59) 1.69 (1.24–2.30)	Age, race, randomized treatment assignment, smoking status, alcohol use, vigorous exercise, history of hypertension, hypercholesterolemia, cardiovascular disease, and diabetes
4	K. Craig Kent *et al.* ^22^	2010	Cohort study	USA	23 446	3 033 009	≤25 (22.3) >25 (27.5)	1.00 (1.00–1.00) 1.20 (1.17–1.22)	Sex, age, race/ethnicity, high-blood pressure, coronary artery disease, family history of AAA, high cholesterol, diabetes, peripheral arterial disease, carotid disease, cerebrovascular history, smoking, quit smoking, fruit & veg, nuts, and exercise
5	Kevin C. Chun *et al.* ^23^	2014	Cohort study	USA	469	5673	<30 ≥30	1.00 (1.00–1.00) 0.94 (0.77-1.15)	Age, total cholesterol, eGFR, HTN, diabetes, CAD, COPD, PVD, statin use, and smoking
6	Daniel R. Wong *et al.* ^24^	2007	Cohort study	USA	376	38976	12.9–23.0 (18.0) 23.1–24.3 (23.7) 24.4–25.7 (25.1) 25.8–27.4 (26.6) 27.5–91.7 (32.6)	1.00 (1.00–1.00) 0.82 (0.55–1.21) 1.15 (0.82–1.61) 1.24 (0.88–1.73) 1.25 (0.89–1.75)	Age, smoking, hypertension, diabetes, hypercholesterolemia, and physical activity
7	Kelli L. Summers *et al.* ^25^	2020	Cohort study	USA	267	9190	18.5–24.9 (21.7) <18.5 (15.3) 25–29.9 (27.5) 30–34.9 (32.5) 35–39.9 (37.5) >40 (42.5)	1.00 (1.00–1.00) 1.07 (0.42–2.70) 1.11 (0.83–1.48) 0.83 (0.54–1.28) 1.05 (0.53–2.09) 2.04 (0.85–4.91)	Age, sex, ethnicity, and medical conditions
8	Eiman Jahangir *et al.* ^26^	2015	Cohort study	USA	158	6528	<25 ≥25	1.00 (1.00–1.00) 0.72 (0.49–1.08)	Sex, race, education, history of smoking, history of MI/CABG, history of high-blood pressure, history of high cholesterol, and history of diabetes
9	Eiman Jahangir *et al.* ^26^	2015	Cohort study	USA	123	11 692	<25 ≥25	1.00 (1.00–1.00) 0.64 (0.40–1.03)	Sex, race, education, history of smoking, history of MI/CABG, history of high-blood pressure, history of high cholesterol, and history of diabetes
10	Linn Nyrønning *et al.* ^27^	2019	Cohort study	Norway	622	58 514	<25 (22.0) 25–29 (27.0) 30–34 (32.0) ≥35 (37.0)	1.00 (1.00–1.00) 0.96 (0.80–1.16) 1.10 (0.87–1.41) 1.19 (0.77–1.84)	Age at HADS-D measure, sex, diabetes mellitus, coronary heart disease, hypertension, total cholesterol, and smoking
11	Toril Rabben *et al.* ^28^	2021	Cohort study	Norway	330	12 480	≤30 >30	1.00 (1.00–1.00) 1.02 (1.00–1.03)	Diabetes mellitus, hypertension, and smoking

**Table 4:** Characteristics of individual studies on BMI and the risk of mortality after AAA repair.

No.	Author	Year	Study type	Country	Patients (*n*)	Surgical repair type	Outcomes	BMI groups (mean)	Relative risk (95% CI) in multivariate regression model	Adjustment for confounders
1	Michael S. Miller *et al.* ^29^	2019	Cohort study	USA	492	EVAR	All-cause mortality on perioperative and postoperative outcomes	18.5–25 (21.8) 25–30 (27.5) 30–35 (32.5)	1.00 (1.00–1.00) 0.61 (0.40–0.92) 0.68 (0.39–1.18)	Age, male, diabetes, CAD, smoking, renal complication, and stroke or TIA
2	Kristina A Giles *et al.* ^30^	2010	Cohort study	USA	2097	OSR	All-cause mortality on perioperative outcomes	18.6–30 (24.3) <18.6 (16.1) 30–35 (32.5) 35–40 (37.5) ≥40 (42.5)	1.00 (1.00–1.00) 0.90 (0.20–3.80) 1.00 (0.60–1.90) 1.00 (0.30–3.00) 2.60 (1.04–6.30)	Age, chronic steroid use, PVD with prior surgery, cardiac disease, and renal disease
3	Kristina A Giles *et al.* ^30^	2010	Cohort study	USA	3358	EVAR	All-cause mortality on perioperative outcomes	18.6–30 (24.3) <18.6 (16.1) 30–35 (32.5) 35–40 (37.5) ≥40 (42.5)	1.00 (1.00–1.00) 3.50 (0.99–12.10) 1.30 (0.60–3.00) 1.40 (0.40–5.20) 2.50 (0.70–8.50)	Age, female sex, and cardiac disease
4	William P. Shutze Sr *et al.* ^31^	2018	Cohort study	USA	336	EVAR	All-cause mortality on 5-year outcomes	25–30 (27.5) <25 (22.5) >30 (32.5)	1.00 (1.00–1.00) 1.72 (1.01–2.94) 0.60 (0.24–1.52)	Sex, age, hypertension, iliac artery length, and aortic neck length
5	Joshua K. Kays *et al.* ^32^	2018	Cohort study	USA	505	OSRand EVAR	All-cause mortality on 30-day postoperative outcomes	18.5–24.9 (21.8) <18.5 (16.0) 25–29.9 (27.5) ≥30 (32.5)	1.00 (1.00–1.00) 5.90 (2.29–15.23) 0.74 (0.47–1.15) 0.56 (0.34–0.92)	Age, and Charlson Comorbidity Index
6	Athanasios Saratzis *et al.* ^33^	2014	Cohort study	UK	159	EVAR	All-cause mortality on postoperative (>30 days) outcomes	<30 (25.0) ≥30 (33.0)	1.00 (1.00–1.00) 1.00 (0.90–1.20)	Hypertension, PAD, and diabetes
7	Timothy C. Huber *et al.* ^34^	2019	Cohort study	USA	407	EVAR	All-cause mortality on perioperative and postoperative outcomes	<18.5 (15.3) 18.5–25 (21.8) 25–30 (27.5) ≥30 (32.5)	1.00 (1.00–1.00) 0.21 (0.08–0.53) 0.23 (0.09–0.57) 0.25 (0.10–0.63)	Age, sex, race, coronary artery disease, diabetes mellitus, hypertension, hyperlipidemia, Charleston Comorbidity Index, statin use at time of AAA repair, statin use after AAA repair, and total psoas muscle area

Two researchers independently rated the quality of the final included studies using the Newcastle–Ottawa Scale (NOS) with scores ranging from 0 to 9 points and any discrepancies were solved via discussing with the corresponding author. Since all our final included studies are cohort studies, the NOS was used to evaluate quality in three aspects: selection, comparability, and outcome, respectively. All entries in the selection and outcome evaluations could be scored 1 if satisfied and 0 if not; entries in the comparability evaluation could be scored up to 2 with a minimum score of 0^35^. The total score we applied using the NOS criteria ranged from 0 to 9 (worst to best). Studies with an overall NOS quality score more than 6 points were considered as high-quality.

### Categorization of BMI and obesity

Studies that reported BMI as a continuous variable were not included in our dose-response analysis. Studies that reported two or more BMI categories with mean values and corresponding OR values were adopted in our dose-response meta-analysis. However, some of the included studies did not report the mean or median BMI values for each category. Hence, the midpoint was assigned as the averaged BMI value for closed BMI interval. For open ended highest category (i.e. 40 kg/m^2^) or lowest category (i.e. 18 kg/m^2^), the boundary was assumed to have the same amplitude as the adjacent category^11^. When a study reported risk estimates relative to a reference category other than the lowest BMI category, the risk estimates were recalculated and adjusted in our meta-analysis using the lowest one as the reference by the Greenland-Longnecker method^36^.

### Statistical analysis

All statistical analyses were performed using StataSE version 15.1 (College Station). The routine meta-analysis of binary variables by STATA meta. Ado module was performed as a way to determine whether there was a relationship between obesity and AAA presence. Due to the low incidence of AAA presence (4.8%, with a 95% CI of 4.3–5.3)^37^, risk ratios (RR) could be treated as ORs in most studies. We compared the ORs and 95% CIs of the highest level of BMI category versus the lowest level of BMI category. Recognizing the substantial variation regarding the participants’ demographics and the confounders for RR among different studies included in our meta-analysis, we assumed that the true effect could vary from study to study due to the potential high heterogeneity among studies. Hence, the random effects model was adopted in our meta-analysis to assess the summarized OR and 95% CI. A two-sided *P*-value <0.05 was considered statistically significant.

Afterward, we attempted to establish a dose-response relationship between BMI and the risk of AAA presence as well as AAA postoperative mortality through the robust-error meta-regression (REMR) model described by Xu and Doi^38^. This is a one-stage method that considers all included studies as a whole while treating each study as a cluster in order to validate the fitting of a linear or nonlinear dose-response curve. In order to considering both linearity and nonlinearity in one model, we used the restricted cubic spline (RCS) function to fit the dose-response trend.

Thereafter, based on the REMR model and by using StataSE 15.1 software, we performed two major dose-response meta-analysis individually to determine the relationship between different BMI values and the risk of developing AAA as well as the relationship between BMI and the risk of AAA postoperative mortality. To be specific, the dose-response meta-analysis by REMR model required BMI-transformed doses (undergone centralization), at least two quantitatively classified BMI categories per study and the corresponding natural log RR values with 95% CI for each BMI-transformed dose. Additionally, a likelihood ratio test was used to assess for the nonlinearity of the dose-response relationship. However, studies with two open ended BMI intervals that did not report mean BMI values were excluded in our analysis. The results of our dose-response meta-analysis were considered statistically significant when *P*-value was less than 0.05. All the dose-response curves were re-visualized and presented by R Studio version 4.2.1. Moreover, statistical heterogeneity was assessed by *Q*-statistic test and I-squared (*I* ^2^) was used to represent the heterogeneity between studies.

To investigate the effect of potential confounders in our dose-response meta-analysis focusing on the association between BMI and the risk of AAA postoperative mortality, we also performed subgroup analyses to further explore whether this association was influenced by the type of postoperative mortality (i.e. short-term and long-term mortality) and the type of surgical repair (i.e. OSR and EVAR). If there were three or more studies and enough data points for RCS curving, subgroup dose-response meta-analyses were performed based on the available characteristics of the studies.

In addition, to judge the robustness of the meta-analysis results, we carried out sensitivity analyses to estimate the stability of our meta-analysis. One study at a time was removed and the remaining studies were analyzed to clarify whether a single study significantly deviated from the study results. Small study effects, such as publication bias, were assessed by Egger’s test and Begg’s test^39,40^. To provide a clear indication of publication bias for the studies included in our meta-analysis, Galbraith plots were generated and shown. An estimate of the 95% confidence range was marked within solid lines, meaning an expectation is that 95% of the analyzed studies fall within this range^41^.

## Results

### Systematic search results, study selection, and quality control

**Figure 1:**
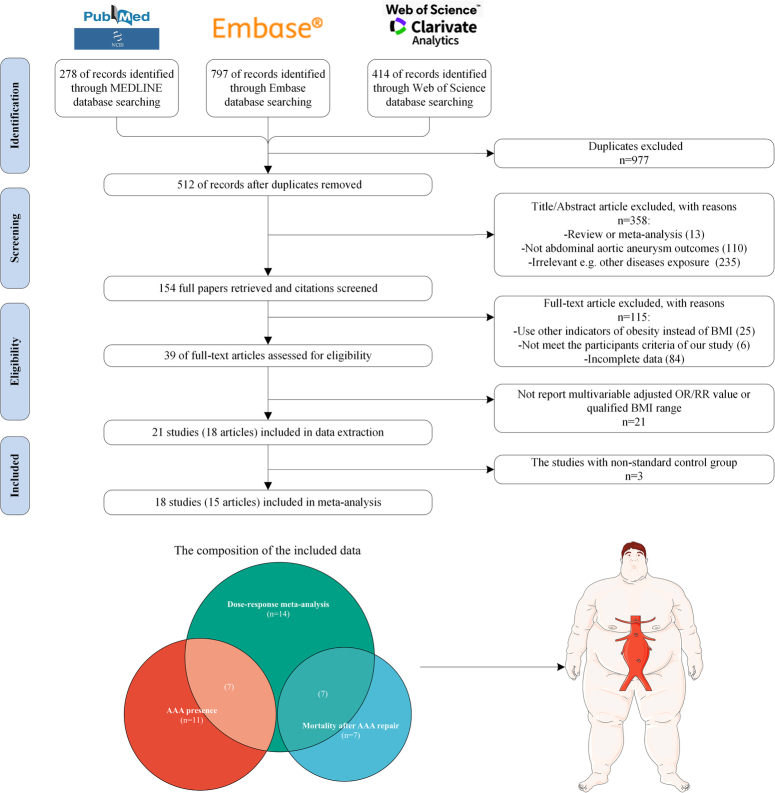
The flowchart showing the process of literature screening and study selection. AAA, abdominal aortic aneurysm; OR, odds ratio; RR, relative risk.

The flowchart showing the process of literature screening and study selection is presented in Figure 1. At last, 18 studies (15 articles) in total were included in our study. Among them, 11 of the 18 studies were integrated to explore the association between obesity and AAA presence and seven of these 11 studies were eligible for the dose-response meta-analysis. Besides, the remaining seven of the 18 studies were utilized to unveil the dose-response relationship between BMI levels and the risk of AAA postoperative mortality.

All the 18 studies involved in this meta-analysis were cohort studies from 15 articles published between the year of 2007 and 2021^20–34^. The NOS quality assessment results of these 18 studies are shown in SDC Table 2 (Supplemental Digital Content 5, http://links.lww.com/JS9/B791), and all of them were scored with at least six stars, which means they are all considered high-quality studies.

### Characteristics of the included studies

The demographic and clinical data of subjects enrolled in 11 selected studies regarding BMI and its association with AAA presence are shown in Table 1. The similar general characteristics of the remaining seven studies evaluating BMI and its quantitative correlation with the mortality after AAA surgical repair are presented in Table 2. The included articles represented a range of geographical areas in Europe (*n*=5) and North America (*n*=13). The involved 18 studies also represent both prospective cohort studies and retrospective cohort study published between 2007 and 2021. The detailed characteristics of the included studies, especially the original data for our subsequent dose-response meta-analysis are shown in Tables 3 and 4.

### Obesity and the risk of AAA presence

In order to explore the association between obesity and the risk of AAA presence, 11 studies included more than 3 228 004 participants were used to evaluate the risk of AAA morbidity in people with the highest BMI levels (defined as obesity) compared with the lowest BMI levels (defined as nonobesity).

**Figure 2:**
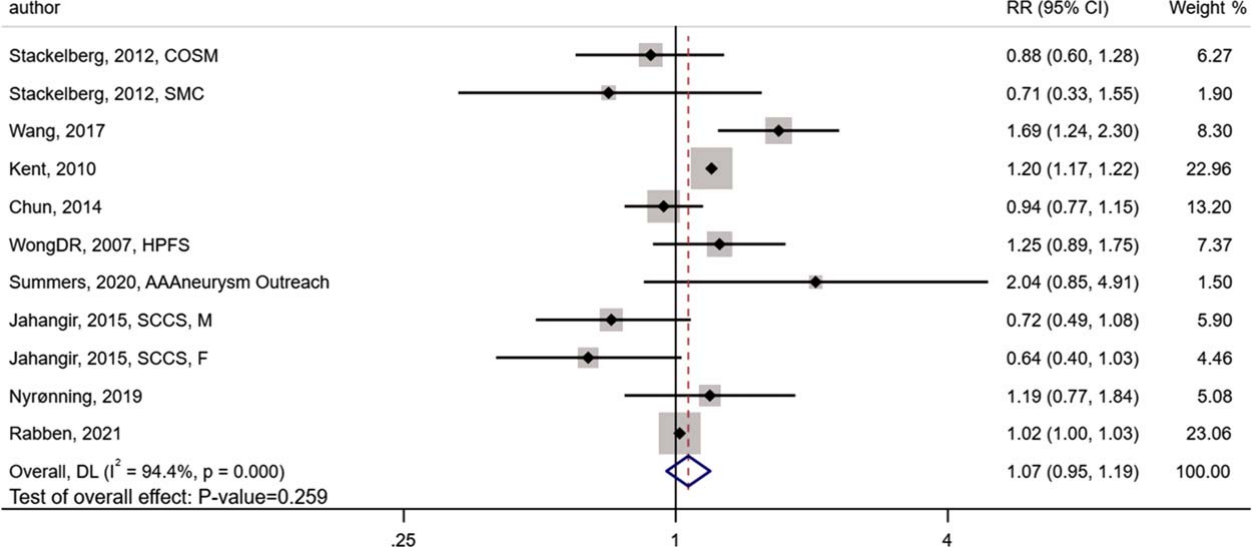
The forest plot showing the summary RR of AAA presence associated with obesity. AAA, abdominal aortic aneurysm; RR, relative risk.

For the meta-analysis results assessed by random effects model, the overall relative risk (RR) of AAA presence associated with obesity was 1.07 (95% CI: 0.95–1.19; *P*-value=0.259) and the statistical heterogeneity assessed by *Q* test showed that *I* ^2^=94.4%, which indicated a very high degree of heterogeneity. The forest plot showing the summarized RR results analyzed by random effects model was presented in Figure 2.

The overall RR (associated with obesity) of AAA presence was greater than 1.00 (zero-risk baseline), meaning that obesity does play a role in AAA morbidity to some degree. However, the random effects model also indicated that the *P*-value of summary RR was greater than 0.05, implying that perhaps there is a more complicated, unexpected relationship between obesity and the risk of AAA presence.

### The dose-response relationship between BMI and the risk of AAA presence

**Figure 3:**
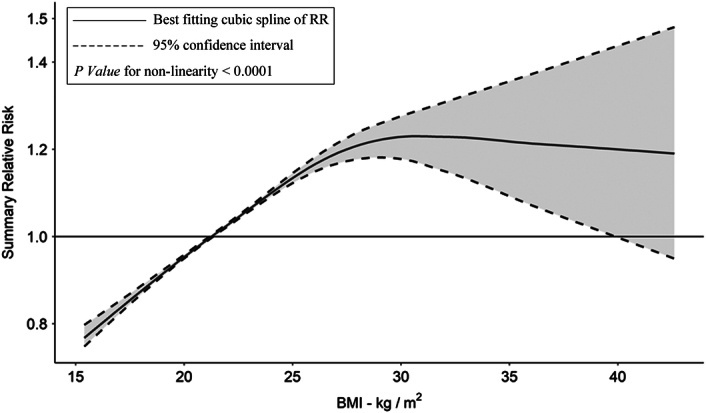
The nonlinear dose-response relationship between BMI and the relative risk of AAA presence. AAA, abdominal aortic aneurysm; RR, relative risk.

To further explore the quantitative relationship between obesity and the risk of AAA presence, we utilized BMI as a measurement for obesity in our subsequent dose-response meta-analysis. Data were extracted from a total of seven cohort studies and input into the REMR model to yield the association between BMI and the risk of AAA presence. Based on the REMR model, there was solid evidence indicating a significant nonlinear relationship between BMI and AAA presence (*P*-value for nonlinearity <0.0001). This nonlinear relationship between BMI and the risk of AAA presence was visualized in Figure 3 and the detailed RRs data with corresponding BMI levels from the nonlinear REMR dose-response analysis were presented in Table 5. To be specific, the RR value of AAA presence increases monotonically and drastically from 0.77 (95% CI: 0.75–0.80) to 1.15 (95% CI: 1.13–1.17) with elevating BMI ranging from 15.4 to 25.6, correspondingly. Afterward, the RR value increases slowly from 1.15 (95% CI: 1.13–1.17) to 1.23 (95% CI: 1.17–1.28) with escalating BMI ranging from 25.6 to 30.4, respectively. Thereafter, the RR value remains at 1.23 with increased BMI and even undergoes a little reduction but is still around 1.19 to 1.23 with a wider range of 95% CI.

**Table 5:** Summary table of relative risks (95% CIs) from the nonlinear REMR dose-response analysis of BMI and the risk of AAA presence.

BMI and the risk of AAA presence						
Model	Nonlinear (restricted cubic spline, RCS)					
Center value	21.8					
Number of knots	3					
knot values	21 27 32					
Number of observations	26					
Number of studies	7					
R-squared	0.807					
Root MSE	0.045					
P for nonlinearity	<0.0001					
	Coefficient (β)	Robust Std. Err.	t	*P-value*>|t|	95% CI	
doses 1 (β1)	1.040898	0.0026022	16.03	0.000	1.034551	1.047285
doses 2 (β2)	0.9722786	0.0056632	−4.83	0.003	0.9585196	0.9862352
cons	0.417386	0.0227834	−16.01	0.000	0.3651999	0.4770294
Levels of BMI (kg/m^2^)	Summary relative risk (RR)		95% CI (lower limit)		95% CI (upper limit)	
15.40	0.77		0.75		0.80	
21.80	1.00		1.00		1.00	
25.60	1.15		1.13		1.17	
26.80	1.19		1.16		1.21	
27.00	1.19		1.17		1.21	
27.55	1.20		1.18		1.23	
28.90	1.22		1.19		1.26	
30.40	1.23		1.17		1.28	
30.45	1.23		1.17		1.28	
31.80	1.23		1.15		1.30	
32.55	1.22		1.14		1.32	
36.40	1.21		1.06		1.38	
36.80	1.21		1.06		1.38	
37.60	1.21		1.04		1.40	
42.60	1.19		0.95		1.48	

### The dose-response relationship between BMI and the risk of postoperative mortality after AAA surgical repair

Next, we further aimed at exploring the quantitative relationship between obesity and the risk of AAA postoperative mortality. We utilized BMI as a parameter for obesity and applied it together with multivariate-adjusted RR values in our dose-response meta-analysis.

**Figure 4:**
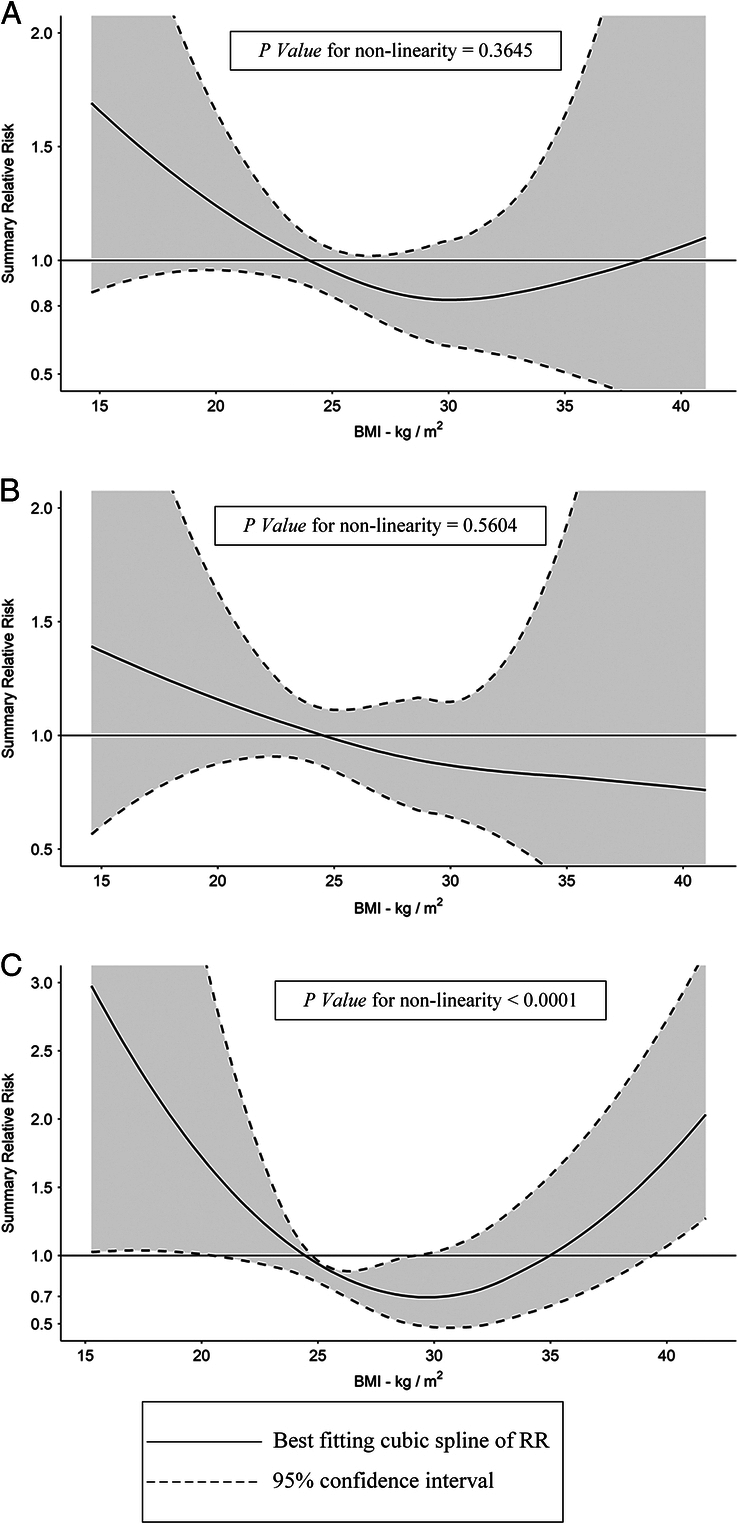
The dose-response association between BMI and the risk of mortality after AAA surgical repair. (A) The dose-response association between BMI and the risk of all-cause mortality after two combined types of AAA surgical repair (EVAR+OSR); (B) The dose-response association between BMI and the risk of all-cause mortality after EVAR treatment of AAA; (C) The dose-response association between BMI and the risk of all-cause short-term mortality after two combined types of AAA surgical repair (EVAR+OSR). AAA, abdominal aortic aneurysm; EVAR, endovascular aneurysm repair; OSR, open surgical repair; RR, relative risk.

Data were extracted from a total of seven relevant cohort studies and input into the REMR model to yield the relationship between BMI and the risk of postoperative mortality after AAA surgical repair. According to the results from REMR model, there was a medium degree of nonlinear relationship between BMI and the risk of all-cause mortality after AAA surgical repair (*P*-value for nonlinearity=0.3645). Overall, the nonlinear relationship could be visualized as a ‘U’ shape curve of RRs with varying BMI levels (Fig. 4A). Therefore, we can observe the minimal value of RR and its corresponding BMI level from the ‘U’ shape curve. The summary table of RR data from the nonlinear REMR dose-response analysis of BMI and the all-cause mortality after AAA surgical repair (OSR+EVAR) were presented in Table 6.

**Table 6:** Summary table of relative risks (95% CIs) from the nonlinear REMR dose-response analysis of BMI and the all-cause mortality after AAA surgical repair (OSR+EVAR).

BMI and the all-cause mortality after AAA surgical repair (OSR+EVAR)						
Model	Nonlinear (restricted cubic spline, RCS)					
Center value	22.85					
Number of knots	3					
knot values	22 28 36					
Number of observations	26					
Number of studies	7					
R-squared	0.182					
Root MSE	0.336					
P for nonlinearity	0.3645					
	Coefficient (β)	Robust Std. Err.	t	*P-value*>|t|	95% CI	
doses 1 (β1)	0.9458571	0.0354147	−1.49	0.188	0.8630515	1.036608
doses 2 (β2)	1.069694	0.0763063	0.94	0.381	0.8983665	1.273695
cons	3.808556	3.35714	1.52	0.180	0.4405914	32.92187
Levels of BMI (kg/m^2^)	Summary relative risk (RR)		95% CI (lower limit)		95% CI (upper limit)	
14.65	1.69		0.88		3.24	
17.05	1.47		0.91		2.38	
17.85	1.41		0.92		2.15	
22.85	1.07		0.98		1.16	
27.85	0.85		0.67		1.08	
28.55	0.83		0.65		1.08	
28.60	0.83		0.64		1.08	
29.35	0.83		0.63		1.08	
30.85	0.83		0.62		1.11	
31.05	0.83		0.61		1.12	
33.55	0.87		0.56		1.35	
33.60	0.87		0.56		1.36	
35.05	0.91		0.50		1.64	
36.05	0.94		0.46		1.89	
40.05	1.06		0.32		3.50	
41.05	1.10		0.29		4.10	

Although the nonlinearity of this dose-response relationship was not significantly strong, the ‘U’ shape curve contained considerable clinical values so we still focused on this nonlinear relationship result. To interpret, the RR of AAA postoperative mortality decreases monotonically and dramatically from 1.69 (95% CI: 0.88–3.24) to 0.83 (95% CI: 0.65–1.08) with elevating BMI ranging from 14.65 to 28.55, respectively, but the slope of the curve diminishes gradually. Afterward, the RR value stabilizes at 0.83 with BMI interval from 28.55 to 31.05, indicating a ‘safest’ BMI interval with the minimal RR for AAA postoperative mortality. Thereafter, the RR value increases again from 0.83 (95% CI: 0.61–1.12) to 1.10 (95% CI: 0.29–4.10) with ascending BMI ranging from 31.05 to 41.05, correspondingly.

In addition, we also analyzed the dose-response relationship between BMI and the RR of AAA postoperative mortality based on linear REMR model and the result was visualized in SDC Figure 3a (Supplemental Digital Content 6, http://links.lww.com/JS9/B792). This result showed a monotonically decreasing tendency of RRs with rising BMI levels.

### Subgroup analyses

We were curious to explore more underlying relationships between BMI and the RRs of different subtypes of AAA postoperative mortality. Hence, based on the available data, we carried out two specific subgroup analyses focusing on the subtype of surgical repair (i.e. EVAR) and the subtype of all-cause mortality (i.e. short-term mortality).

The subgroup dose-response meta-analysis focusing on BMI and the all-cause mortality after AAA EVAR included five cohort studies and revealed a weak nonlinear relationship between these two variables (*P*-value for nonlinearity=0.5604). Overall, this nonlinear relationship can be depicted as a monotone decline curve with a gradually flattening out tail (Fig. 4B). The summary table of RR data from the nonlinear REMR dose-response analysis of BMI and the all-cause mortality after AAA EVAR therapy were presented in Table 7. Besides, the linear dose-response relationship between BMI and the RR of AAA post-EVAR mortality were yielded by linear REMR model and was visualized in SDC Figure 3b (Supplemental Digital Content 6, http://links.lww.com/JS9/B792).

**Table 7:** Summary table of relative risks (95% CIs) from the nonlinear REMR dose-response analysis of BMI and the all-cause mortality after AAA endovascular aneurysm repair (EVAR).

BMI and the all-cause mortality after AAA endovascular aneurysm repair (EVAR)						
Model	Nonlinear (restricted cubic spline, RCS)					
Center value	22.77					
Number of knots	3					
knot values	22 28 33					
Number of observations	17					
Number of studies	5					
R-squared	0.146					
Root MSE	0.314					
P for nonlinearity	0.5604					
	Coefficient (β)	Robust Std. Err.	t	*P-value*>|t|	95% CI	
doses 1 (β1)	0.9667582	0.0488592	−0.67	0.540	0.8401907	1.112392
doses 2 (β2)	1.014486	0.0852179	0.17	0.872	0.8034483	1.280955
cons	2.278414	2.66398	0.70	0.520	0.088672	58.5435
Levels of BMI (kg/m^2^)	Summary relative risk (RR)		95% CI (lower limit)		95% CI (upper limit)	
14.57	1.39		0.59		3.26	
17.77	1.25		0.73		2.13	
22.77	1.06		0.98		1.14	
27.77	0.91		0.67		1.23	
28.52	0.89		0.65		1.21	
29.27	0.88		0.65		1.18	
30.77	0.86		0.63		1.16	
30.97	0.85		0.62		1.17	
33.52	0.83		0.45		1.54	
34.97	0.82		0.35		1.92	
35.97	0.81		0.29		2.25	
39.97	0.77		0.14		4.37	
40.97	0.76		0.11		5.17	

The subgroup dose-response meta-analysis focusing on BMI and the all-cause short-term mortality after AAA surgical repair (OSR+EVAR) consisted of three cohort studies and indicated a strong nonlinear relationship between these two variables (*P*-value for nonlinearity <0.0001). Overall, this nonlinear relationship also shows an ‘U’ shape curve of RRs with varying BMI levels (Fig. 4C). At the beginning stage of the curve, the RR of AAA postoperative short-term mortality declines monotonically and sharply from 2.99 (95% CI: 1.04–8.60) to 0.69 (95% CI: 0.49–0.98) with escalating BMI ranging from 15.27 to 29.17, respectively. The optimum BMI level corresponding to the minimal RR value (0.69) equals to 29.17. However, afterward, the RR value turns into an upward trend from 0.69 (95% CI: 0.49–0.98) to 2.03 (95% CI: 1.27–3.23) with increasing BMI levels ranging from 29.17 to 41.67, correspondingly. The summary table of RR data from the nonlinear REMR dose-response analysis of BMI and the all-cause short-term mortality after AAA surgical repair (OSR+EVAR) were presented in Table 8.

**Table 8:** Summary table of relative risks (95% CIs) from the nonlinear REMR dose-response analysis of BMI and the all-cause short-term mortality after AAA surgical repair (OSR+EVAR).

BMI and the all-cause short-term mortality after AAA surgical repair (OSR+EVAR)						
Model	Nonlinear (restricted cubic spline, RCS)					
Center value	23.47					
Number of knots	3					
Knot values	23 30 34					
Number of observations	14					
Number of studies	3					
R-squared	0.527					
Root MSE	0.413					
P for nonlinearity	<0.0001					
	Coefficient (β)	Robust Std. Err.	t	*P-value*>|t|	95% CI	
doses 1 (β1)	0.8877987	0.0482792	−2.19	0.160	0.7025829	1.121841
doses 2 (β2)	1.121572	0.0321183	4.01	0.057	0.9915526	1.268641
cons	18.41886	25.20171	2.13	0.167	0.051111	6637.598
Levels of BMI (kg/m^2^)	Summary relative risk (RR)		95% CI (lower limit)		95% CI (upper limit)	
15.27	2.99		1.04		8.60	
17.67	2.25		1.01		5.01	
23.47	1.13		0.93		1.37	
29.17	0.69		0.49		0.98	
31.67	0.73		0.47		1.14	
34.17	0.93		0.59		1.46	
36.67	1.20		0.76		1.90	
41.67	2.03		1.27		3.23	

### Sensitivity analysis and small study effects

The results of the sensitivity analysis on our meta-analyses demonstrated in Result 3.3 and 3.4 were showed in SDC Figure 1c (Supplemental Digital Content 7, http://links.lww.com/JS9/B793, 2c, Supplemental Digital Content 8, http://links.lww.com/JS9/B794), respectively. Omitting the studies of Wang *et al*.^21^ and Kent *et al*.^22^ significantly modified the pooled risk estimates in sensitivity analysis. The sensitivity analysis results of our meta-analyses involved in Result 3.5 and 3.6 were showed in SDC Figure 4c (Supplemental Digital Content 9, http://links.lww.com/JS9/B795, 5c, Supplemental Digital Content 10, http://links.lww.com/JS9/B796 and 6c, Supplemental Digital Content 11, http://links.lww.com/JS9/B797). Among them, sensitivity analysis by excluding the studies of Kays *et al*.^32^ and Huber *et al*.^34^ significantly altered the pooled RR estimates, suggesting the results may be unstable.

There were no significant publication bias and no indication for small study effects according to the funnel plots by the Begg’s tests or the Egger’s tests for all those associations (SDC Figure 1a-b, Supplemental Digital Content 7, http://links.lww.com/JS9/B793, 2a-b, Supplemental Digital Content 8, http://links.lww.com/JS9/B794, 4a-b, Supplemental Digital Content 9, http://links.lww.com/JS9/B795, 5a-b, Supplemental Digital Content 10, http://links.lww.com/JS9/B796, 6a-b, Supplemental Digital Content 11, http://links.lww.com/JS9/B797). However, for BMI and the risk of short-term mortality after AAA surgical repair, the Galbraith plot indicated between-study variability due to the values outside of the 95% confidence limits (SDC Figure 6d, Supplemental Digital Content 11, http://links.lww.com/JS9/B797). The other analysis results showed by Galbraith plots were presented in SDC Figure 1d (Supplemental Digital Content 7, http://links.lww.com/JS9/B793, 2d, Supplemental Digital Content 8, http://links.lww.com/JS9/B794, 4d, Supplemental Digital Content 9, http://links.lww.com/JS9/B795, 5d, Supplemental Digital Content 10, http://links.lww.com/JS9/B796).

## Discussion

AAA is a common vascular disease, which results from the chronic local inflammation in aortic wall and is significantly associated with perivascular adipose tissue (PVAT)^42^. The presence of AAA may also lead to severe cardiovascular and cardiopulmonary complications or further contribute to the ruptured AAA and even the mortality. However, previous studies and clinical cases have shown inconsistent and controversial data about the relationship between obesity and AAA, which raises problems in the prevention, treatment, and prognosis of AAA. In order to solve the ‘obesity paradox’ in AAA, our systematic review and dose-response meta-analysis for the first time determined the quantitative association between BMI and the risk of AAA presence and postoperative mortality, providing valuable clinical guidance for the prevention and therapy of AAA.

First of all, the routine meta-analysis of obesity and the risk of AAA presence concluded that the population with obesity has a 7% increased risk of AAA morbidity compared to people without obesity. However, a lack of statistical significance (*P*-value >0.05) as well as the high heterogeneity (*I* ^2^=94.4%) implied a more complicated underlying relationship between obesity and AAA presence, motivating us to further explore the dose-response quantitative relationship between them.

Secondly, the dose-response meta-analysis focusing on BMI and AAA presence unveiled a nonlinear positive correlation. The RR value of AAA presence increases monotonically and drastically from 1.00 to 1.19 with elevating BMI ranging from 21.8 to 26.8, indicating this BMI interval (BMI=21.8–26.8) is the key in controlling the risk of AAA morbidity. Even a little rise of BMI within this ‘risky interval’ may lead to significant increase of risk of AAA presence. According to this finding, paying more attention to the population with 21.8≤BMI≤26.8 and promoting the frequency of AAA screening in this population may be of great significance for public health.

Thirdly, the dose-response meta-analysis focusing on BMI and the risk of mortality after AAA surgical repair revealed a ‘U’ shape curve showing a nonlinear association. The ‘U’ shape curve illustrates that with the ascending of BMI levels, the RR of postoperative mortality goes down at the beginning and reaches the minimal RR, but then gradually goes up and restores high levels of RR. According to the minimal RR (RR=0.83), we can yield its corresponding BMI levels and define 28.55≤BMI≤31.05 as the ‘safest’ BMI interval. Therefore, considering the combinational AAA surgical repair methods (EVAR+OSR), perhaps it will be helpful to reduce the postoperative mortality if AAA patients can control their BMI within or close to this interval. This result may be explained from two aspects: (1) Low BMI is usually an indication of frailty and asthenia in clinical practices, especially while considering aged population. The frailty and asthenia in elderly people commonly elevate the risks of postoperative complications and relapse, which may eventually result in the high risk of postoperative mortality^43–45^. (2) High BMI is commonly related to the high risk of other chronic diseases including hyperlipidemia, hypertension, and hypercholesterolemia, which are closely associated with atherosclerosis (AS). Hence, AAA patients with morbid obesity may suffer from higher risk of mortality after surgical repair and longer mean length of hospital stays^45^. To sum up, suggesting AAA patients control their BMI in an appropriate interval [28.55, 31.05] is reasonable for those who choose surgical repair as a treatment plan.

Last but not the least, subsequent subgroup analysis pointed out a similar ‘U’ shape relationship between BMI and the risk of short-term mortality after AAA surgical repair. The nonlinearity of this relationship is even more evident and the optimum BMI level equals 29.17, corresponding to the minimal RR value (RR=0.69). The optimum BMI value still falls in the category of overweight, which suggests that even though weight loss strategies should be considered for AAA patients affected by obesity with an anticipation of surgical repair procedures, maintaining a BMI level that is slightly higher than the category of ‘normal weight’ is equally beneficial for the postoperative rehabilitation and the reduction of short-term mortality after surgery (EVAR+OSR). Inversely, as for the association between BMI and the risk of mortality after EVAR, it shows a negative correlation instead of a ‘U’ shape correlation. Although this result may be limited by small sample size and low certainty of evidence, it can still partially demonstrate the view that EVAR may serve as a more feasible treatment option than OSR for patients suffering from obesity in combination with AAA^46^.

The pathogenesis of AAA is complex, involving genetic, epigenetic, and environmental factors. Among them, we infer that PVAT, a central player in the pathogenesis and progression of AAA, may be highly associated with the ‘obesity paradox’ in AAA. The function of PVAT in artery has a duality: on the one hand, PVAT plays a protective role on the vascular wall, secreting various chemokines, and adipocytokines around vessels to maintain vascular homeostasis in normal physiological conditions; on the other hand, PVAT loses its normal function and promotes the progression of vascular diseases by mediating inflammation responses in pathological conditions^42,47^. Thus, we hypothesize that appropriate PVAT density may act as a protective factor for vascular homeostasis but excessive density of PVAT will cause the dysregulation of the inflammatory responses in vessels and finally increase the risk of AAA presence or the postoperative mortality^48^. Additionally, investigation of the PVAT transcriptome in AAA patients has revealed substantial differences in PVAT gene expression pattern, indicating PVAT plays a central role in the regulatory network of AAA involving inflammatory genes, miRNAs, cytokines, and function cells^47,49,50^. Overall, PVAT may provide a plausible potential mechanism of the ‘obesity paradox’ in AAA but future studies are still needed to further illustrate this issue.

### Strengths and limitations

Our meta-analysis has several strengths. To our knowledge, this is the first systematic review and dose-response meta-analysis assessing the quantitative relationship between BMI and the risk of AAA presence and postoperative mortality. The strength of our study is also reflected in the inclusion of more updated literature, a large number of participants and the multivariate-adjusted RR data, which make the results more reliable and convincing. In addition, the analytical method utilized in our study was the REMR model, also known as the ‘one-stage’ model. Compared with previous dose-response meta-analyses using Generalized Least Squares (GLS) method as a ‘two-stage’ model, the REMR method eliminates the bias generated by the GLS method, resulting in a better error estimation, wider study applicability and better fit to the data^38^. Besides, our meta-analysis included prospective cohort studies, which can effectively reduce the possibility of a reverse relation, the risk of recall and selection bias, enhancing the possibility of an etiological hypothesis. At last, all the main results were relatively robust, examined by sensitivity analysis, Begg’s test, Egger’s test, and Galbraith plots.

Although the traditional categorical meta-analysis and dose-response analysis were reasonably and simultaneously applied in our systematic review, it has several potential limitations. First, most of our included studies only focused on European/American population so the potential bias from this perspective was inevitable. Second, there was a high heterogeneity among the included studies but we failed to perform more subgroup analyses based on other confounders like sex, age, and region due to the lack of data. Finally, it was a pity that we did not perform dose-response meta-analysis focusing on BMI and AAA growth/progression due to the lack of relevant research. However, overall, the results of this study were reliable and of great importance for clinical guidance.

## Conclusions

This systematic review demonstrates that obesity or BMI is independently associated with the risk of AAA presence but this positive correlation shows a nonlinear quantitative characteristic. In the dose-response analysis, we also find that BMI is related to AAA postoperative mortality in a ‘U’ shaped pattern, with the lowest relative risk observed among patients with BMI ranging from 28.55 to 31.05. This ‘U’ shape association is even more evident in the present subgroup analysis regarding BMI and the short-term mortality. Overall, our research is of great significance for the prevention of AAA morbidity and improving the prognosis of AAA patients undergone surgical repair. Certainly, future studies with large sample sizes and well-designed groupings are needed to strengthen the evidence. In addition, further investigations remain necessary to delineate the underlying pathophysiological mechanisms of the ‘obesity paradox’ among patients with AAA and to provide clinical guidance.

## Ethics approval, consent to participate, and consent to publish

Ethical approval was given in every original study.

## Consent

Not applicable.

## Sources of funding

This work was supported by the Fundamental Research Funds for the Central Universities (Grant Number: DUT22YG107), the National Natural Science Foundation of China (Grant Number: 81600370), and the China Postdoctoral Science Foundation (Grant Number: 2018M640270) for Yanshuo Han; This work was also supported by the National Natural Science Foundation of China (Grant Number: 81970402) for Jian Zhang.

## Author contribution

Y.H. and J.Z.: conceived and designed this study; Y.W. and H.Z.: searched the databases; Y.W., H.Z., F.Y., and Y.H.: conducted the meta-analysis, analyzed the data, and drafted the manuscript; Y.W., P.G., and X.Z.: checked all the data; D.J. and J.Z.: contributed to reviewing and revising the paper. All authors read and approved the final manuscript.

## Conflicts of interest disclosure

The authors have no relevant financial or nonfinancial interests to disclose.

## Research registration unique identifying number (UIN)

1. Name of the registry: Prospero.
2. Unique identifying number or registration ID: CRD42021262816.
3. Hyperlink to this specific registration: https://www.crd.york.ac.uk/prospero/display_record.php?ID=CRD42021262816.

## Guarantor

Yanshuo Han, MD. School of Life and Pharmaceutical Sciences, Dalian University of Technology, Panjin, Liaoning Province, People’s Republic of China. E-mail: yanshuohan@dlut.edu.cn Tel.: +86 427 2631410.

## Data availability statement

The datasets used and/or analyzed during the current study are available from the corresponding author on reasonable request.

Availability of data and materials: The datasets used and/or analyzed during the current study are available from the corresponding author on reasonable request.

## Provenance and peer review

Not commissioned, externally peer-reviewed.

## Supplementary Material

SUPPLEMENTARY MATERIAL
