# Supplementary figures and images for: Body mass index and the risk of abdominal aortic aneurysm presence and postoperative mortality: a systematic review and dose-response meta-analysis
Source: Int J Surg. 2024 Feb 5;110(4):2396–410. doi: 10.1097/JS9.0000000000001125 (PMC11020033; doi:10.1097/JS9.0000000000001125)

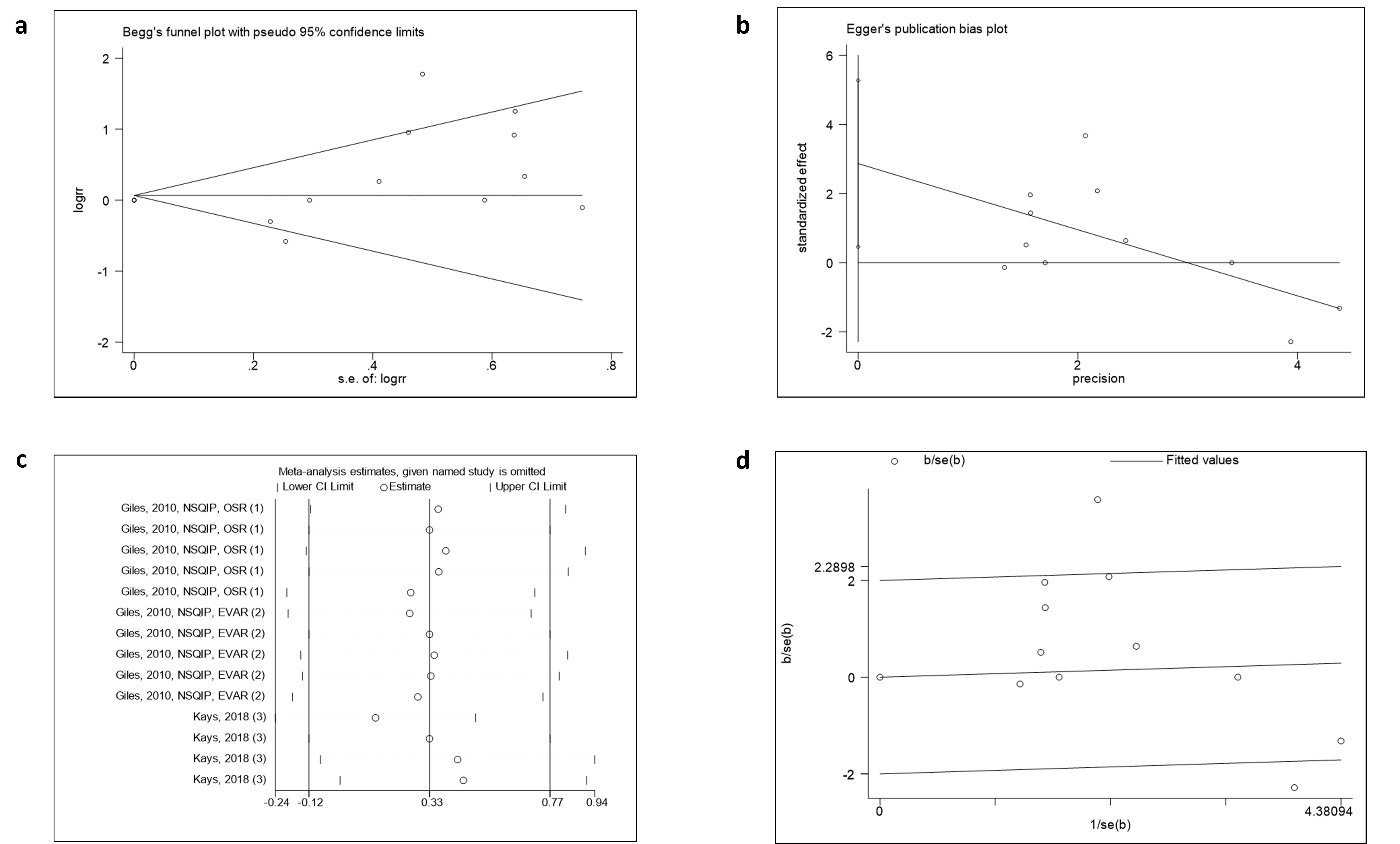

Supplement: SUPPLEMENTARY MATERIAL [file js9-110-2396-s002.jpg]

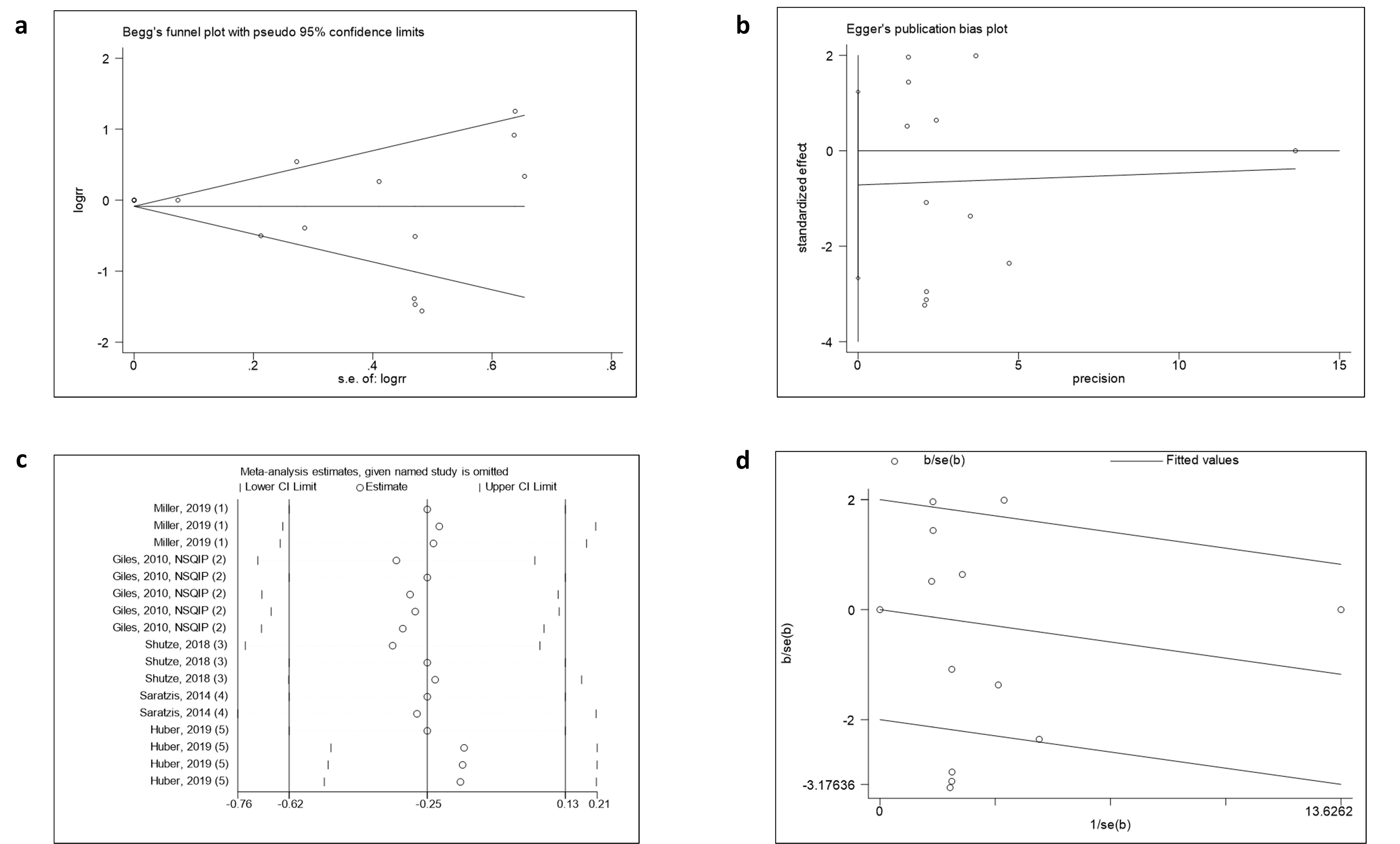

Supplement: SUPPLEMENTARY MATERIAL [file js9-110-2396-s003.jpg]

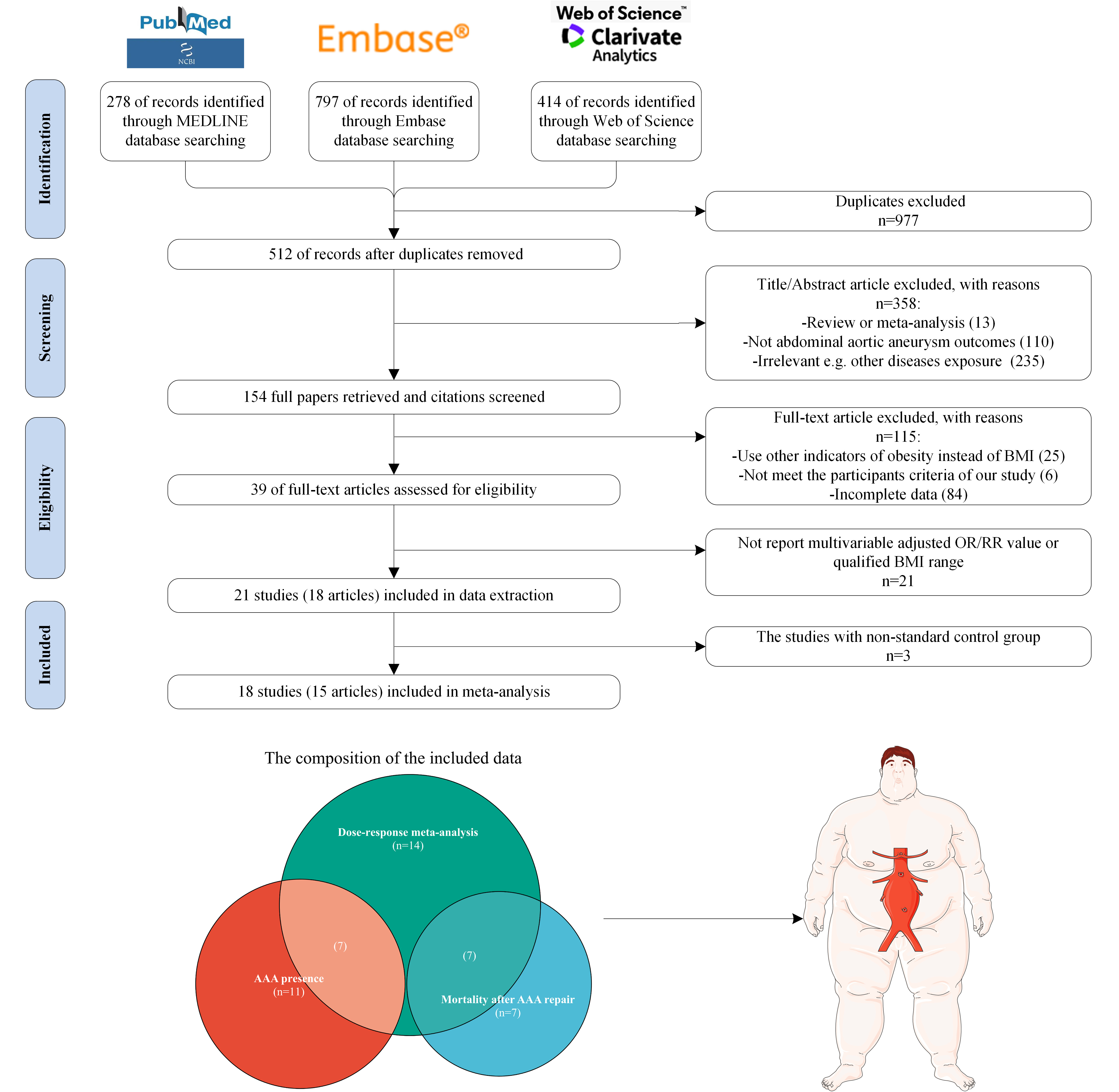

Supplement: SUPPLEMENTARY MATERIAL [file js9-110-2396-s004.jpg]

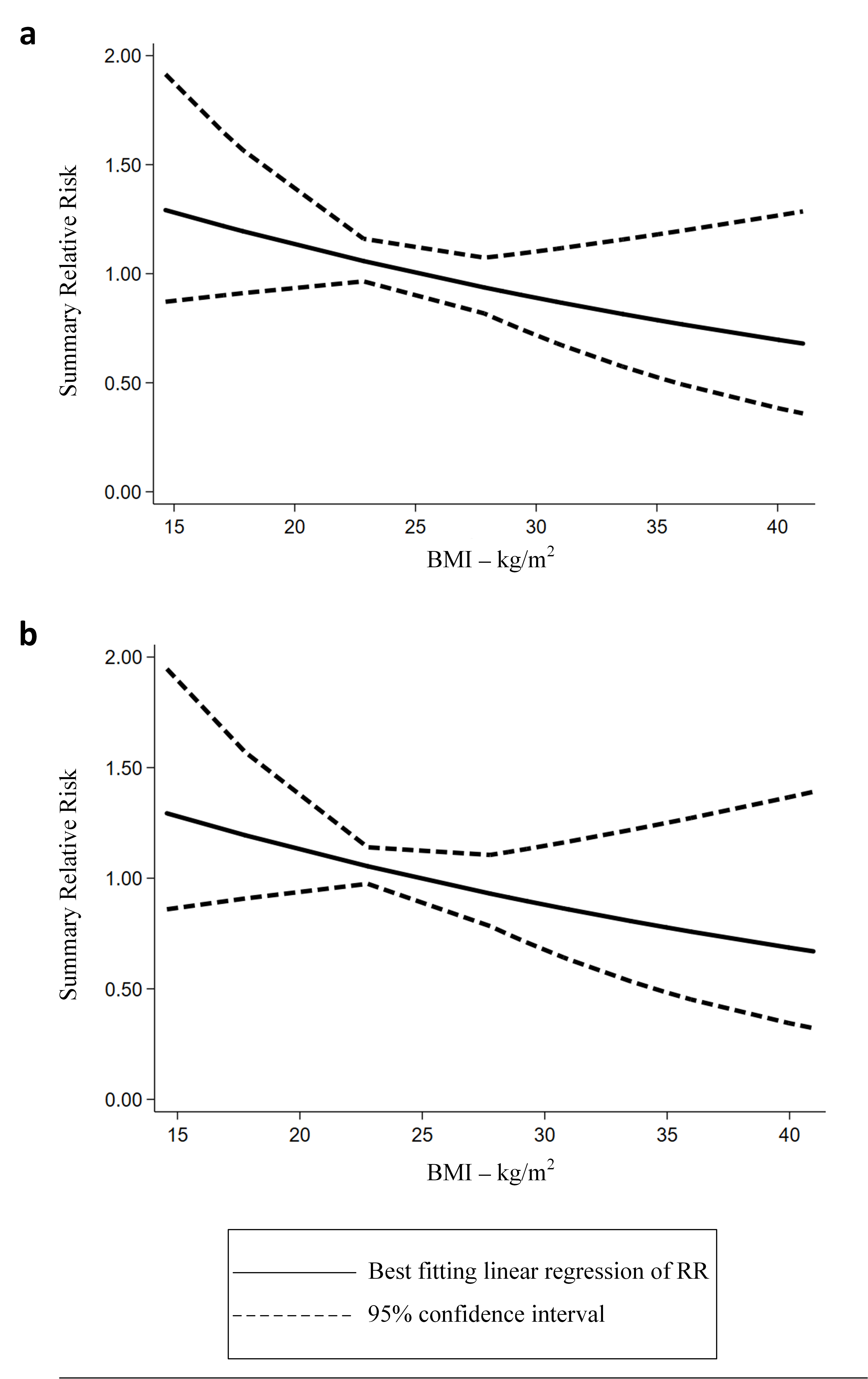

Supplement: SUPPLEMENTARY MATERIAL [file js9-110-2396-s008.jpg]

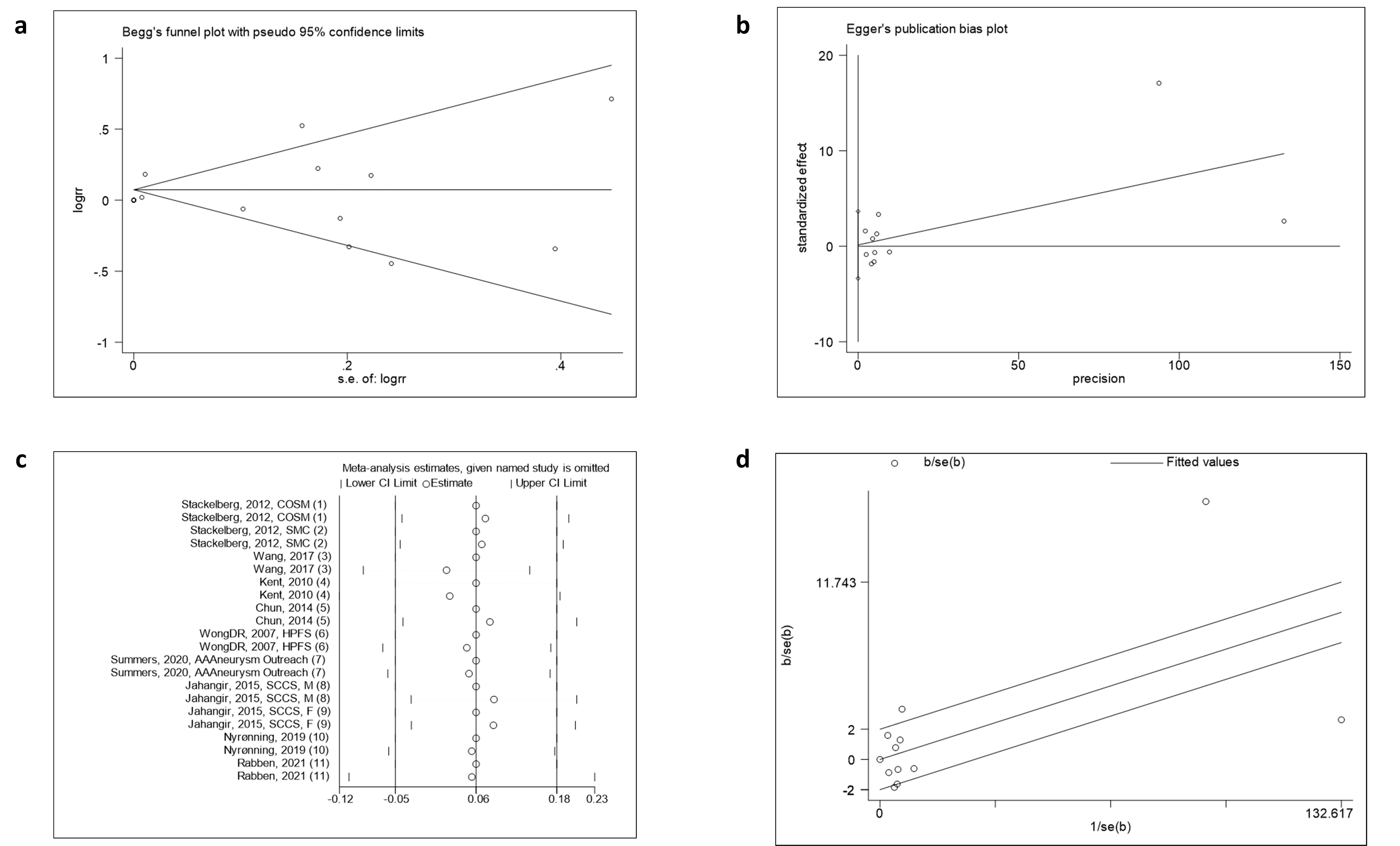

Supplement: SUPPLEMENTARY MATERIAL [file js9-110-2396-s009.jpg]

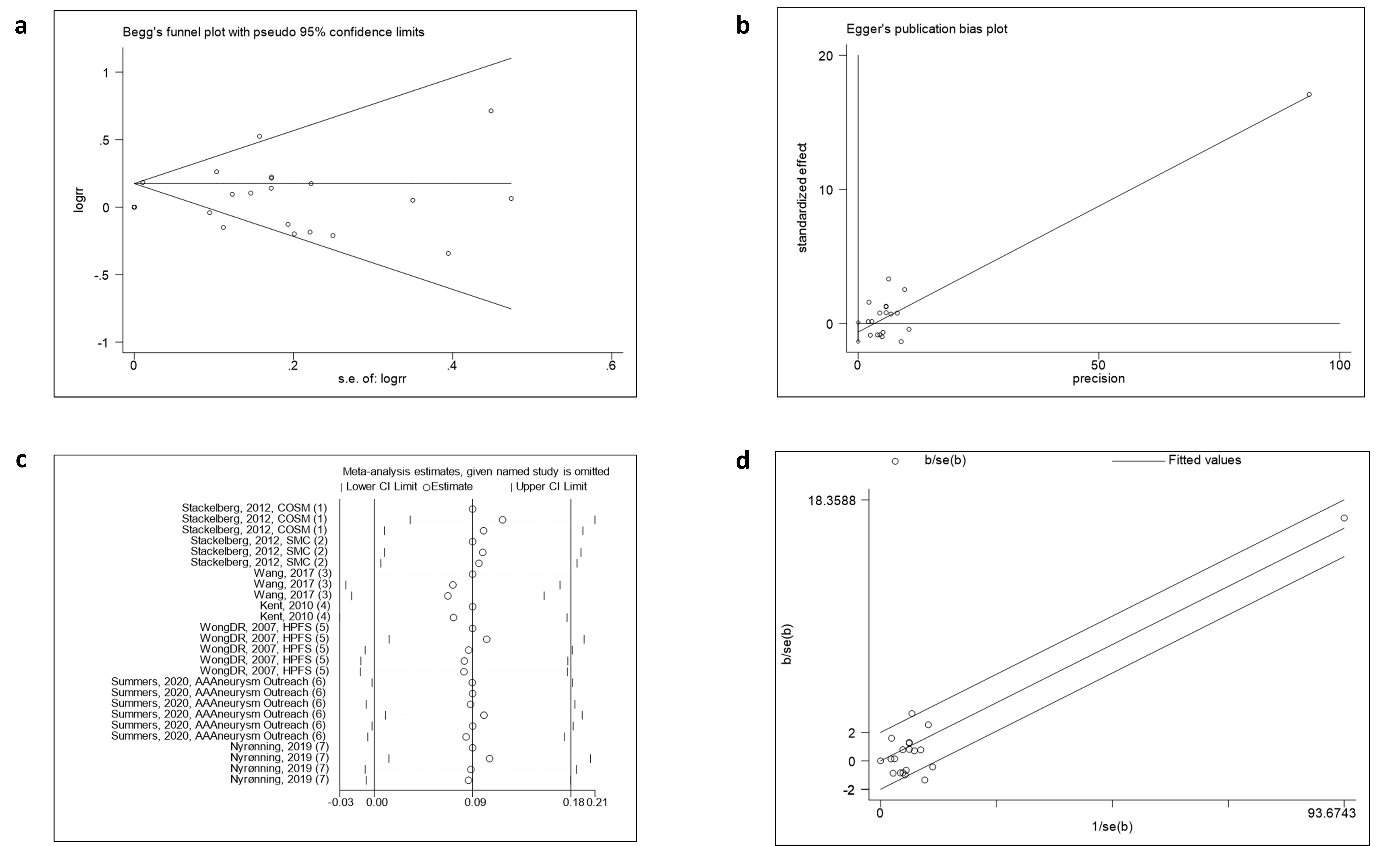

Supplement: SUPPLEMENTARY MATERIAL [file js9-110-2396-s010.jpg]

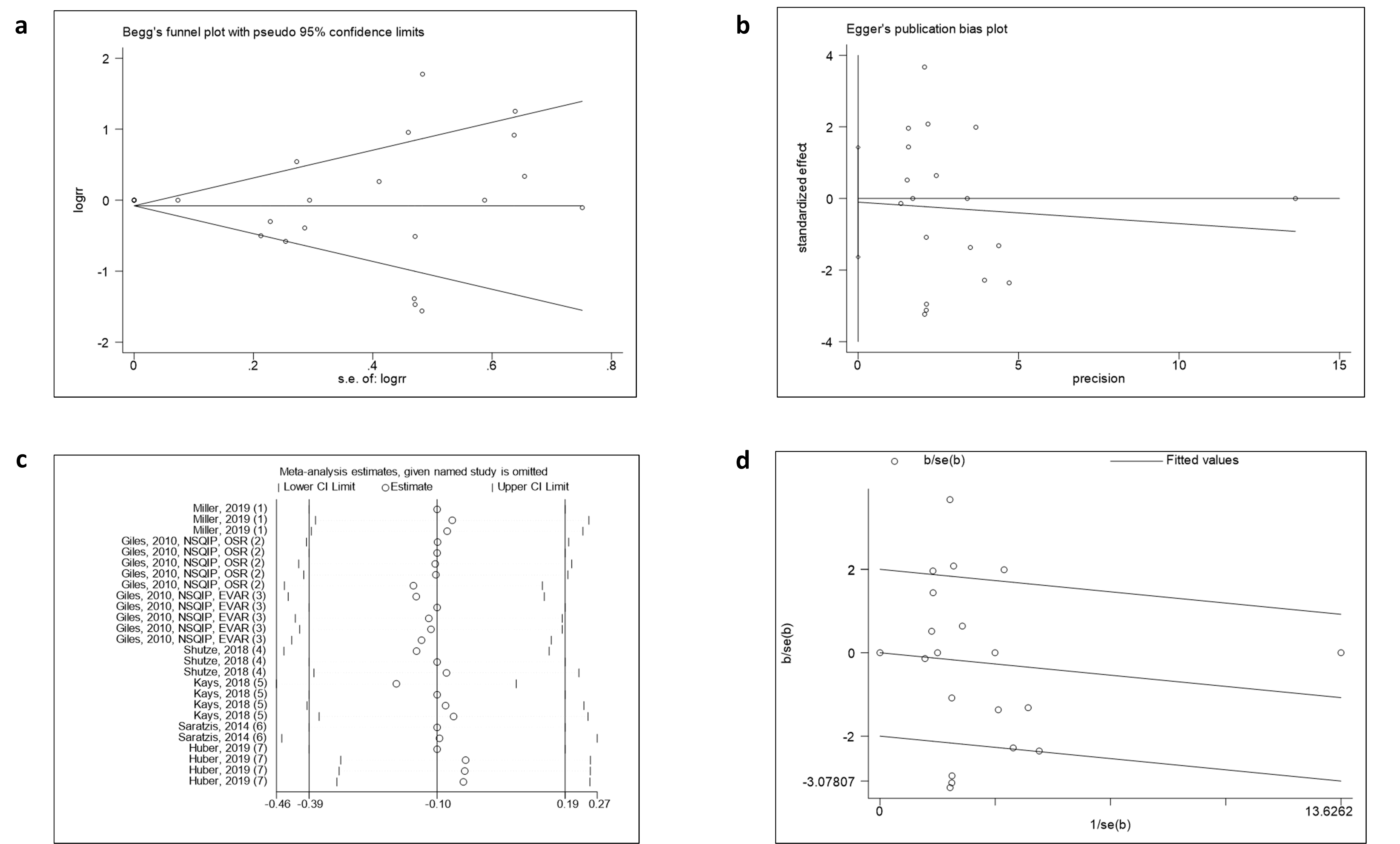

Supplement: SUPPLEMENTARY MATERIAL [file js9-110-2396-s011.jpg]
