# Supplementary material for: Body mass index and the risk of abdominal aortic aneurysm presence and postoperative mortality: a systematic review and dose-response meta-analysis
Source: Int J Surg. 2024 Feb 5;110(4):2396–410. doi: 10.1097/JS9.0000000000001125 (PMC11020033; doi:10.1097/JS9.0000000000001125)
Supplement: SUPPLEMENTARY MATERIAL [file js9-110-2396-s006.pdf]

Supplementary Table S1. Search Strategy.

Studies that reported on the relationship between body mass index (BMI) and abdominal aortic aneurysm (AAA) were selected, which specifically included the association between BMI and AAA presence, as well as BMI and the risk of mortality after AAA surgical repair. Our search combined keywords and MeSH terms. The search strategies for all of the three databases were as following:

1. PubMed:
((body mass index) OR (BMI) OR (obesity) OR (obese) OR (overweight) OR (underweight) OR (asthenia)) AND ((abdominal aortic aneurysm) OR (AAA) OR (aortic aneurysm) OR (aortic disease) OR (vascular disease) OR (artery disease) OR (endovascular repair) OR (EVAR) OR (open surgical repair) OR (OSR) OR (open aneurysm repair) OR (OAR))

2. Embase:
body mass index OR (BMI) OR (obesity) OR (obese) OR (overweight) OR (underweight) OR (asthenia) OR (AAA) OR (aortic disease) OR (vascular disease) OR (artery disease) OR (endovascular repair) OR (EVAR) OR (open surgical repair) OR (OSR) OR (open aneurysm repair) OR (OAR) AND (abdominal aortic aneurysm)

3. Web of Science:
(ALL=(body mass index) OR ALL=(BMI) OR ALL=(obesity) OR ALL=(overweight) OR ALL=(underweight) OR ALL=(asthenia)) AND (ALL=(abdominal aortic aneurysm) OR ALL=(AAA) OR ALL=(aortic aneurysm) OR ALL=(aortic disease) OR ALL=(vascular disease) OR ALL=(artery disease) OR ALL=(endovascular repair) OR ALL=(EVAR) OR ALL=(open surgical repair) OR ALL=(OSR) OR ALL=(open aneurysm repair) OR ALL=(OAR))
