# Supplementary material for: Body mass index and the risk of abdominal aortic aneurysm presence and postoperative mortality: a systematic review and dose-response meta-analysis
Source: Int J Surg. 2024 Feb 5;110(4):2396–410. doi: 10.1097/JS9.0000000000001125 (PMC11020033; doi:10.1097/JS9.0000000000001125)
Supplement: SUPPLEMENTARY MATERIAL [file js9-110-2396-s007.pdf]

Supplementary Table S2. Newcastle-Ottawa Scale (NOS) of All Included Cohort Studies.

No.	Study	Years	SELECTION				COMPARABILITY	OUTCOME			NOS overall scores
			Representativeness of exposed cohort	Selection of the non-exposed cohort	Ascertainment of exposure	Outcome of interest was not present	Comparability of exposure and non-exposure	Ascertainment of outcome	Follow up enough for outcome	Adequacy of follow-up	
1&2	O. Stackelberg *et al*.	2012	★	★		★	★★	★	★	★	8
3	Lu Wang *et al.*	2017		★		★	★★		★	★	6
4	K. Craig Kent *et al.*	2010	★	★		★	★★		★	★	7
5	Kevin C. Chun *et al.*	2014		★	★	★	★★	★	★	★	8
6	Daniel R. Wong *et al.*	2007	★	★		★	★★	★	★	★	8
7	Kelli L. Summers *et al.*	2020	★	★		★	★★	★			6
8&9	Eiman Jahangir *et al.*	2015	★	★	★	★	★★	★			7
10	Linn Nyrønning *et al.*	2019	★	★		★	★★	★	★	★	8
11	Toril Rabben *et al.*	2021	★	★		★	★★	★			6
12	Michael S. Miller *et al.*	2019	★	★	★	★	★★	★			7
13&14	Kristina A Giles *et al.*	2010	★	★	★	★	★★	★	★		8
15	William P. Shutze Sr *et al.*	2018	★	★	★	★	★★	★	★		8
16	Joshua K. Kays *et al.*	2018		★	★	★	★	★	★		6
17	Athanasios Saratzis *et al.*	2014	★	★	★	★	★★	★		★	8
18	Timothy C. Huber *et al.*	2019	★	★	★	★	★★	★		★	8
